# Biosensing by WGM Microspherical Resonators

**DOI:** 10.3390/s16060905

**Published:** 2016-06-17

**Authors:** Giancarlo C. Righini, Silvia Soria

**Affiliations:** 1 Museo Storico della Fisica e Centro Studi e Ricerche Enrico Fermi, 00184 Roma, Italy; 2 Istituto di Fisica Applicata Nello Carrara, CNR, 50019 Firenze, Italy; s.soria@ifac.cnr.it

**Keywords:** photonics, biosensing, microresonator, whispering gallery modes, microsphere

## Abstract

Whispering gallery mode (WGM) microresonators, thanks to their unique properties, have allowed researchers to achieve important results in both fundamental research and engineering applications. Among the various geometries, microspheres are the simplest 3D WGM resonators; the total optical loss in such resonators can be extremely low, and the resulting extraordinarily high *Q* values of 10^8^–10^9^ lead to high energy density, narrow resonant-wavelength lines and a lengthy cavity ringdown. They can also be coated in order to better control their properties or to increase their functionality. Their very high sensitivity to changes in the surrounding medium has been exploited for several sensing applications: protein adsorption, trace gas detection, impurity detection in liquids, structural health monitoring of composite materials, detection of electric fields, pressure sensing, and so on. In the present paper, after a general introduction to WGM resonators, attention is focused on spherical microresonators, either in bulk or in bubble format, to their fabrication, characterization and functionalization. The state of the art in the area of biosensing is presented, and the perspectives of further developments are discussed.

## 1. Introduction

From the 1960s on, the term optical cavity was immediately been associated with a laser device. In the last few decades, however, the quest for miniaturization and integration has led to the development of optical microcavities, namely structures that enable the confinement of light in microscale volumes, which are becoming more and more important in a number of applications other than “simple” lasers. In the process of reducing the size from the classical Fabry–Perot resonator, other geometries and principles have emerged, resulting in novel classes of microcavities, the most important ones being the whispering gallery mode (WGM) resonators and the photonic crystals [1,2,3]. The principle of WGM resonators, in reality, is not new at all, but dates back to 1910–1912 when John William Strutt (Lord Rayleigh) studied the characteristics of the so-called whispering gallery, namely the circular gallery running around the interior of the dome of St Paul’s Cathedral in London [4]. According to Rayleigh’s observations and to the later studies (see, for instance, [5]), it was proven that structures having circular symmetry may sustain the so-called whispering gallery modes that can be interpreted as acoustic or electromagnetic waves that circulate and are strongly confined within the structure. In terms of geometric optics, the confinement is described by the optical rays which are totally internally reflected and focused by the surface itself. Due to minimal reflection losses and to potentially very low material absorption (e.g., in dielectric materials), these resonators can reach exceptionally high quality factors *Q* = *λ*/Δ*λ* (where *λ* is the wavelength at which a resonance occurs and Δ*λ* the linewidth of the resonant wavelength), up to 10^11^, compared to values around 10^5^ for the best Fabry–Perot resonators. To be more precise, a theoretical analysis indicated values of *Q* close to 10^5^ for ideal lossless photonic-bandgap Fabry–Perot resonators, reducing to *Q* > 2000 for real dielectric materials [6]. The ultra-high *Q* values of WGM resonators lead to very high energy density (of the order of GW/cm^2^), very narrow resonant-wavelength lines (<100 KHz at 1550 nm) and a lengthy cavity ringdown: these properties make these structures of great interest for lasers, optical and RF communications, quantum optics and electrodynamics and sensing [7,8,9].

**Figure 1:**
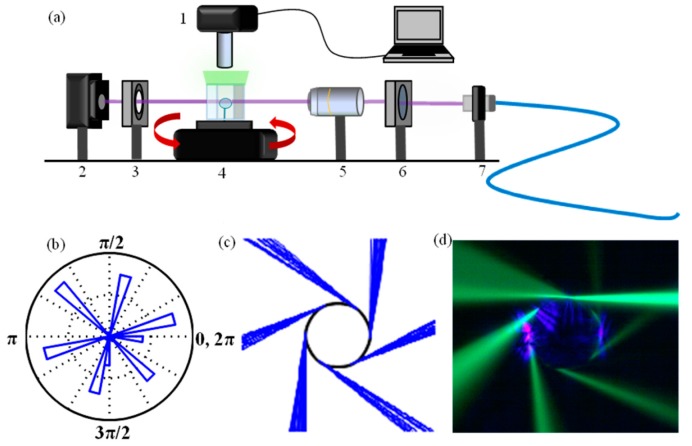
Experimental set-up for visualizing the directional emission of an asymmetric resonant cavity (ARC) in aqueous solution. (**a**) Setup components: 1. imaging system; 2. photodetector; 3. spatial filter; 4. rotation stage, with ARC and the chamber with GFP in PBS buffer; 5. 10× objective; 6. quarter-wave plate for polarization control; 7. fiber collimator; (**b**) Polar histogram of refractive escape for six-pole boundary ARC; (**c**) Ray-tracing simulation; (**d**) Real color image of directional emission by GFP fluorescence imaging of an ARC with a 63-μm diameter. Reprinted with permission from [16].

Since the basic geometric requirement, *i.e.*, circular symmetry, is quite easy to fulfill, several different WGM structures may be considered and have actually been successfully tested, in both two-dimensional (e.g., ring resonators) and three-dimensional shapes; the latter ones include cylindrical (optical fibers, microcapillaries, microdisks, microtoroids, etcetera) and spherical (microspheres and microbubbles) structures, as well as more complex ones (droplets, microbottles, etcetera) [7]. The above requirement, however, is not compulsory, and for instance, non-circularly symmetric WGM cavities, such as 2D racetrack resonators and 3D elliptical resonators, have been developed, which may have the advantage of an easier coupling of the light thanks to a longer interaction length and/or of a directional emission [10,11]. A class of asymmetric resonant cavities (ARC) has also been proposed, in which substantial deformation from cylindrical or spherical symmetry leads to partially chaotic ray dynamics [12]. Thus, even free space coupling is possible in such asymmetric structures, where mode matching of a focused Gaussian beam allows an efficient excitation of high-*Q* WGMs through a process called “chaos-assisted dynamical tunneling” [12,13,14,15,16]. Figure 1 clearly shows, together with the schematic of the experimental setup and the simulation results, the asymmetric directional emission pattern thanks to the fluorescence at ≈509 nm from the green fluorescent protein (GFP) in a phosphate-buffered saline (PBS) solution surrounding the deformed microsphere [16]. Here, the slight deformations to the sphere were produced by a series of 10–20 ms pulses from a focused 30 W CO_2_ laser. Typically, the spheres were pulsed two to three times on two opposite sides. The achieved directional emission makes possible “stand-off” biodetection, where coupling optics can be placed far away from the microresonator and measurements can be made in the far field.

The literature on WGM resonators, thanks to the big scientific efforts in the last decade, is now quite vast, and in this paper, we limit ourselves to providing an overview of recent progresses in a specific field, namely biosensing, achieved by exploiting the properties of a specific structure, namely bulk and hollow microspheres.

## 2. WGM Resonators

**Figure 2:**
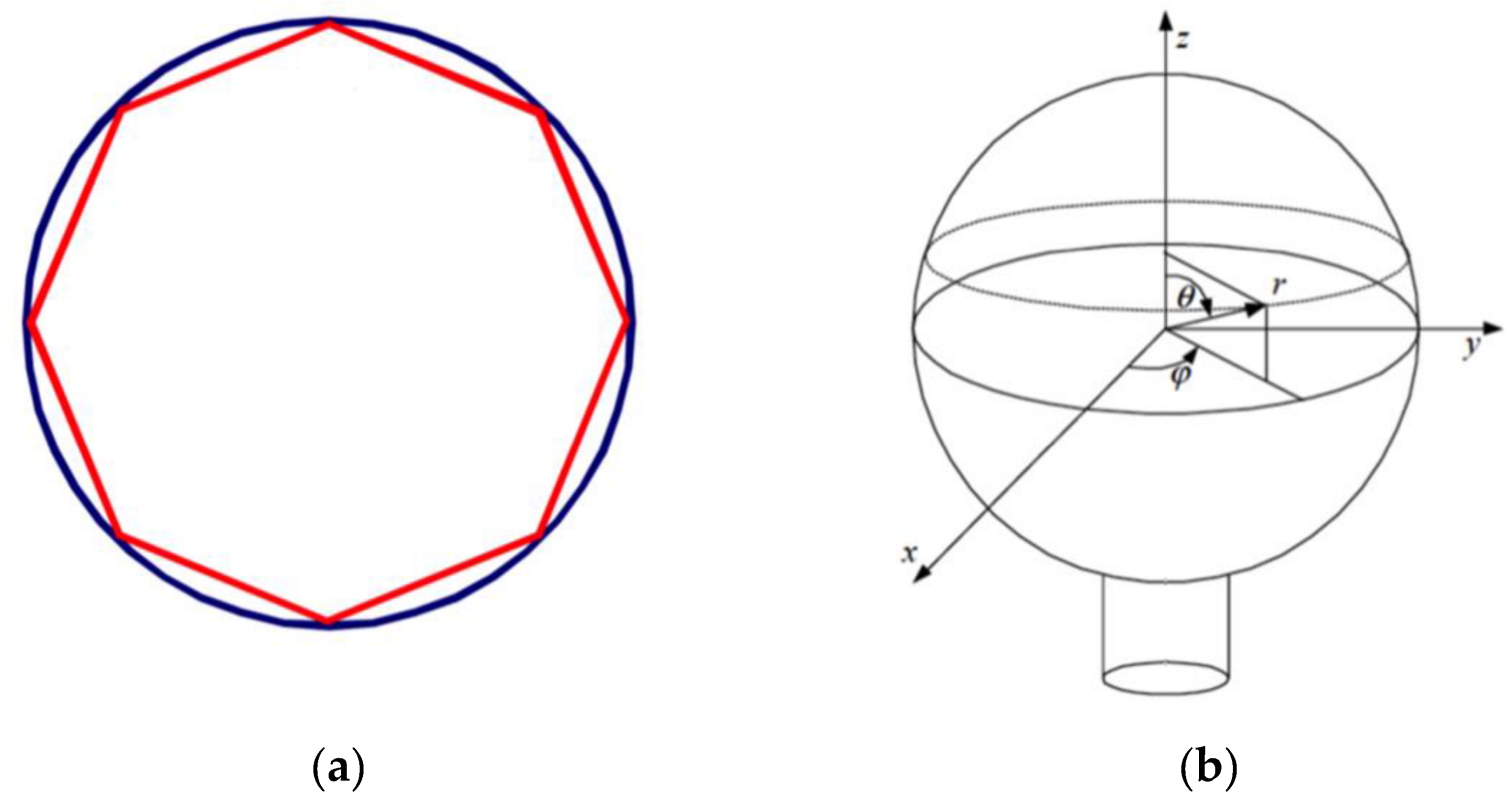
(**a**) WGM supported on the total internal reflection of an optical wave; (**b**) Spherical coordinates in a WGM spherical resonator.

Optical cavities with a high quality factor (*Q*) are used to confine and store light for a length of time by total internal reflections. The light confined inside the cavity is in phase with itself after one round trip, resulting in resonant modes called whispering gallery modes. Sometimes, these modes are also referred to as optical-morphology-dependent resonances (MDRs) [17]. The trapped light propagates around the sphere equator in a narrow and thin band, constantly reflecting off the sphere surface at glancing incidence (see Figure 2).

A first, quick analysis of the propagation can be carried out simply by using geometrical optics. A ray of light will undergo total internal reflection if the angle of incidence *i* is higher than the critical angle $i_{c}$ = arcsin($\frac{1}{N}$), where *N* is the refractive index. A dimensionless size parameter is generally introduced, defined as x = 2 *π a*/*λ* = k *a*, where k is the wavenumber and *a* indicates the radius of the sphere. Let us suppose that the radius of the sphere is much larger than the wavelength of radiation (*a* » *λ* or x » 1) and that rays are at glancing incidence (*i* = *π*/2): at the sphere surface, the condition to have resonance is that the optical path length, which should correspond to an integer number of wavelengths in order to keep in phase, is approximately equal to the circumference of the sphere:

(1) 2πNa=lλ

with *l* the integer number. In this way, it is easy to understand that light may be confined in a band around a great circle of the sphere and that a caustic region can be defined, comprised between the outer sphere and the inner sphere to which the propagating and bouncing rays are tangent. Equation (1) shows clearly that changes to either the radius of the cavity or the refractive index of the optical mode will change the resonant wavelength. The resonant wavelength changes can be written as:

(2) $$\frac{\Delta \lambda }{\lambda_{0}}=\frac{\Delta R_{0}}{R_{0}}+\frac{\Delta N_{s}}{N_{s}}$$

that states that any small change in the refractive index or in the radius must be balanced by a small change in the resonant wavelength.

Quite obviously, the geometrical optics description has severe limitations: as an example, it cannot explain how the light can couple into a WGM (or escape from a WGM) in a perfect sphere, nor can it take into account the polarization of light. A complete description can be provided by the electromagnetic theory, and the resonances can be analyzed using the generalized Lorenz–Mie theory [17]. A detailed description of the mathematical theory can be found in our previous review papers [18,19,20] and also in [21]. The optical modes of a dielectric microsphere can be calculated by solving the Helmholtz equation in spherical coordinates. Polarization is assumed to be constant along the optical trajectories when the microspheres are made of a homogeneous dielectric, and the optical modes reflect with grazing incidence at the dielectric-air boundary. The fields can be expressed in terms of either transverse magnetic (TM) or transverse electric (TE)mode polarization, and solutions are found by solving the scalar equation by the separation of variables approach. The radial field can be described by spherical Bessel functions inside the sphere and an exponential tail outside, while the polar component follows an associated Legendre function, and the equatorial behavior is sinusoidal. A given WGM is identified by mode numbers *n*, *m* and *l* and by the polarization state (TE or TM). The value of *n* gives the number of maxima in the radial component; *l* depends on the equatorial length, expressed as the number of wavelengths; and *l*−|*m*|+1 gives the maxima in the polar component. Polar modes are often referred to as even or odd, based on number of lobes. The presence of an evanescent field tail at the microsphere boundary explains the possibility not only to excite the WGMs inside the spherical microresonator by means of suitable coupling systems, but also their use as biochemical sensors.

One of the most important parameters of microspherical resonators in sensing applications is their quality factor *Q*. It can be considered as an indication of the fraction of the light lost during each cycle around the sphere. The intrinsic *Q* of a microsphere is determined by the contributions from several types of losses: intrinsic curvature losses, scattering losses on residual surface inhomogeneities, intrinsic material losses and, finally, the losses introduced by surface contaminants. The *Q* factor can be mathematically described as the product of the photon lifetime inside the cavity for a given resonant wavelength or the ratio of the resonant wavelength to the spectral linewidth of the resonance:

(3) $$Q=2\pi \nu \tau =\frac{\delta \lambda }{\lambda }$$

In low-loss fused-silica microspheres, with diameters in the range of 50–500 micrometers, *Q*’s in excess of 10^10^ have been demonstrated [22]. It is worth noting that with such high *Q* factors, thermal fluctuations and spectroscopic noise are a real challenge for all WGM resonators-based sensors.

### 2.1. Different Types of WGM Resonators

WGM resonators can be classified according to their geometries and include spheres, toroids, bottles, bubbles, disks, rings, cylinders and capillaries. These can be fabricated using different materials, such as glass, quartz, silicon, silicon-on-insulator (SOI), silicon nitride, polymers and crystals. Common properties of WGM sensors are high *Q* factors, tunability, high stability and sensitivity and the small sample volume needed to detect a given analyte. They can be considered a miniaturized and updated version of the devices used for conventional optical cavity ringdown spectroscopy (CRDS), where long and bulky Fabry–Perot resonators are used to obtain an effective optical path length sufficient to enable high resolution.

Microspherical resonators are extremely simple to fabricate, can reach extremely high *Q* factors and were the first WGMRs tested as biosensors. The details about the fabrication of microspheres in various materials will be provided in the following section (Section 3). Microring and microdisk resonators have lower *Q* factors than other WGMRs, but can be easily fabricated in planar arrays using lithographic or imprinting technologies; single-mode port waveguides for in- and out-coupling the light to and from the resonator can be implemented in the same fabrication process. Integrated WGMRs have been fabricated using a variety of materials, namely silicon oxide [23], silicon nitride [24], silicon-on-insulator (SOI) [25], polymers [26] and porous silicon [27], and also in shapes, such as rings, disk, racetrack rings [28], goblets [29] and edged-disks [30]. Recently, *Q* factors up to 10^7^ have been demonstrated with extremely low loss waveguides in Si_3_N_4_ [31] or in SOI [32]; exceptionally small footprint devices, with disks of a few microns in diameter, have been fabricated, as well. As an example, ultrasmall microring and microdisk lasers with an asymmetric air/GaAs/Al_0.98_Ga_0.02_As waveguide and an active region based on InAs/InGaAs/GaAs quantum dots emitting around 1.3 μm were fabricated and studied, having ultra-small diameters (down to 1 μm) [33,34]. A very important feature of integrated WGMRs is the high multiplexing capability, which is very limited in discrete WGMRs, such as, for instance, microspheres.

Another type of WGM microcavity is constituted by a toroidally-shaped silica cavity supported by a silicon pillar on a microelectronic chip [35]. The toroidal shape of these resonators allows an extra level of geometric control with respect to spherical cavities. Their fabrication often uses oxide-coated Si wafers, and the process includes photolithography, wet and dry etching and laser reflowing. The laser reflowing is used to create a very smooth surface, as it effectively removes lithographic flaws; in this way, a *Q* as high as 4 × 10^8^ @ *λ* =1550 nm was achieved, together with a very small mode volume ≈ 180 μm^3^ [36]. Hybrid silica-polymer ultra-high-*Q* microtoroids were also made, by first lithographically fabricating the silica microtoroid device and then applying a PMMA or polystyrene film; *Q* factors in excess of 10^7^ were measured [37]. Compared to other WGMRs, microtoroids can be fabricated in planar arrays, but their coupling system still remains external to the array, reducing the multiplexing capabilities. Toroidal microcavities appear suitable for strong-coupling cavity quantum electrodynamics (QED) [38]. Microcapillary resonators, also known as liquid core optical ring resonators (LCORR) [39], confine light along their perimeter. They are fabricated by tapering a capillary whose walls have been reduced by fluoridric acid etching. Such WGMRs easily combine photonics with microfluidics, allowing one to physically separate the optical layer from the sensing layer. As an example of sensing applications, Zamora *et al.* reported an LCORR with sub-micron wall thickness, exhibiting improved refractive index sensing [40]. Another very interesting cavity geometry, which makes the integration of microfluidics with the optical sensing device easier, is the hollow microsphere or microbubble [41]. Microbubbles, as LCORRs, separate the fluid layer from the optical layer, warranting no alignment disruption between the resonator and the coupling system during the flow of the analyte; their *Q* factor, however, is larger than LCORRs.

### 2.2. Coupling Systems

**Figure 3:**
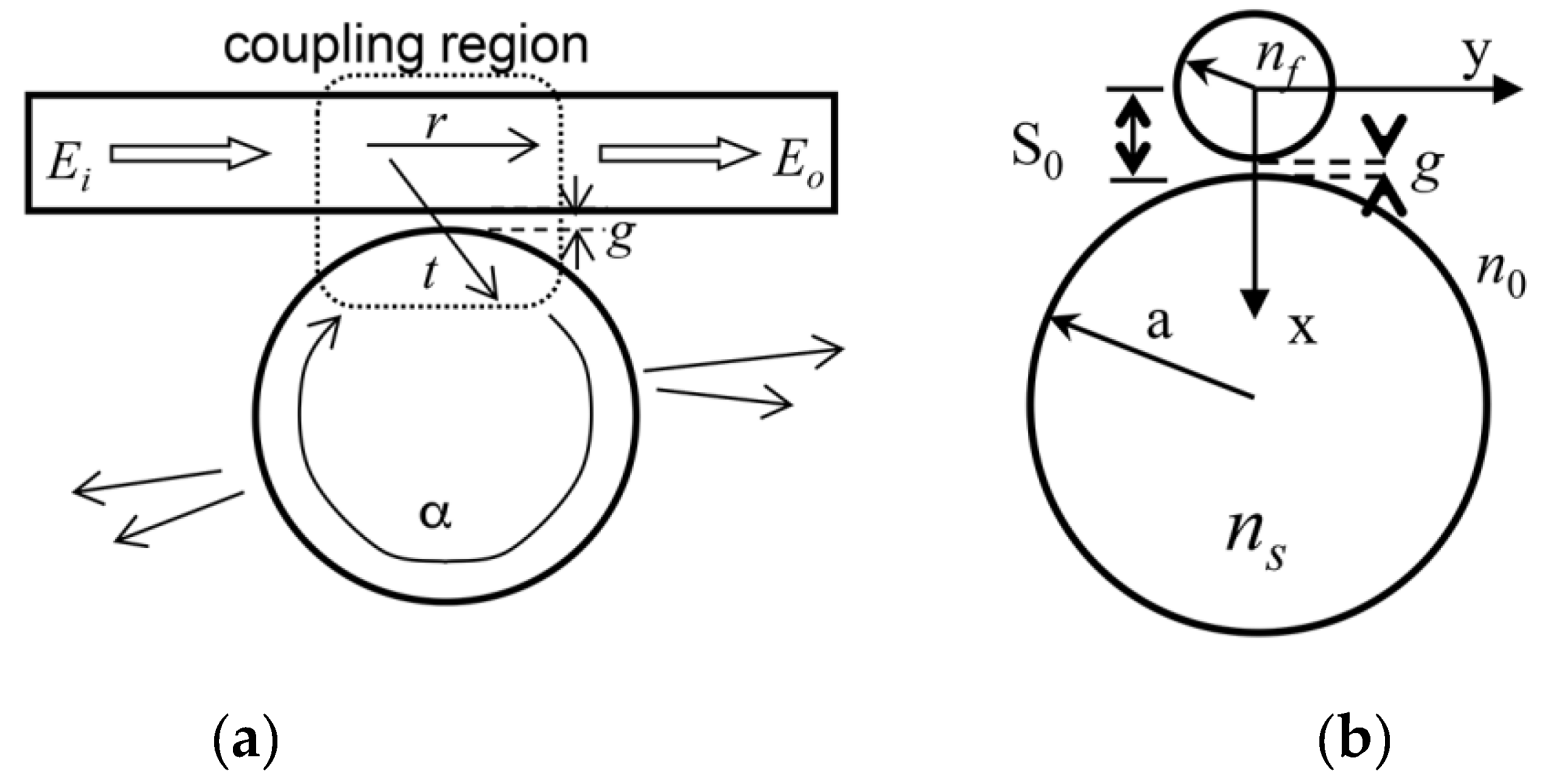
Scheme of a waveguide/fiber to microsphere coupling system: (**a**) lateral and (**b**) cross-sectional view. Reprinted with permission from [42].

The capability of exploiting the unique properties of high-*Q* WGM resonators for fundamental studies and for practical applications effectively depends on the implementation of an efficient and controllable coupling of the light to the cavity modes. The only approach that fulfills these operational principles is based on phase-matched evanescent field coupling. This technique requires an overlap of the evanescent field of the WGM with the evanescent field of the coupler, and an effective excitation of the mode is possible when the phase-matching conditions are also satisfied. Figure 3 shows a general scheme of a waveguide/fiber to microsphere coupling system.

**Figure 4:**
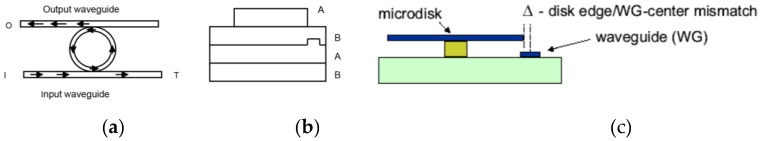
Sketch of the two basic geometries for optically-integrated WGM sensors: (**a**) top view of a microring with two port waveguides: O, output port; I, input port; T, through port; (**b**) cross-section of a microdisk with vertical coupling with a buried waveguide (A Si_3_N**_4_** layer, B SiO_2_ layer); (**c**) cross-section of a microdisk with vertical coupling, non-buried waveguide.

WGM resonators can also be classified according to their degree of planar integration: optically-integrated WGMRs (in-/out-coupling system and cavities are both integrated), hybrid WGMRs (either the cavity or the in-/out-coupling is integrated) and non-integrated systems (neither the cavity nor the in-/out-coupling system is integrated). Generally, in biosensing applications, optically-integrated WGMRs are preferable, because they are more rugged and can be batch manufactured. Usually, microrings or microdisks with two adjacent single-mode port waveguides for coupling the light to and from the resonator are quite convenient; the port waveguides can be arranged horizontally or vertically. In the first case, both waveguides and the WGM sensor are exposed to the analyte, whereas in the second one, the waveguides are shielded from the analyte by a separation layer [24] (see Figure 4).

**Figure 5:**
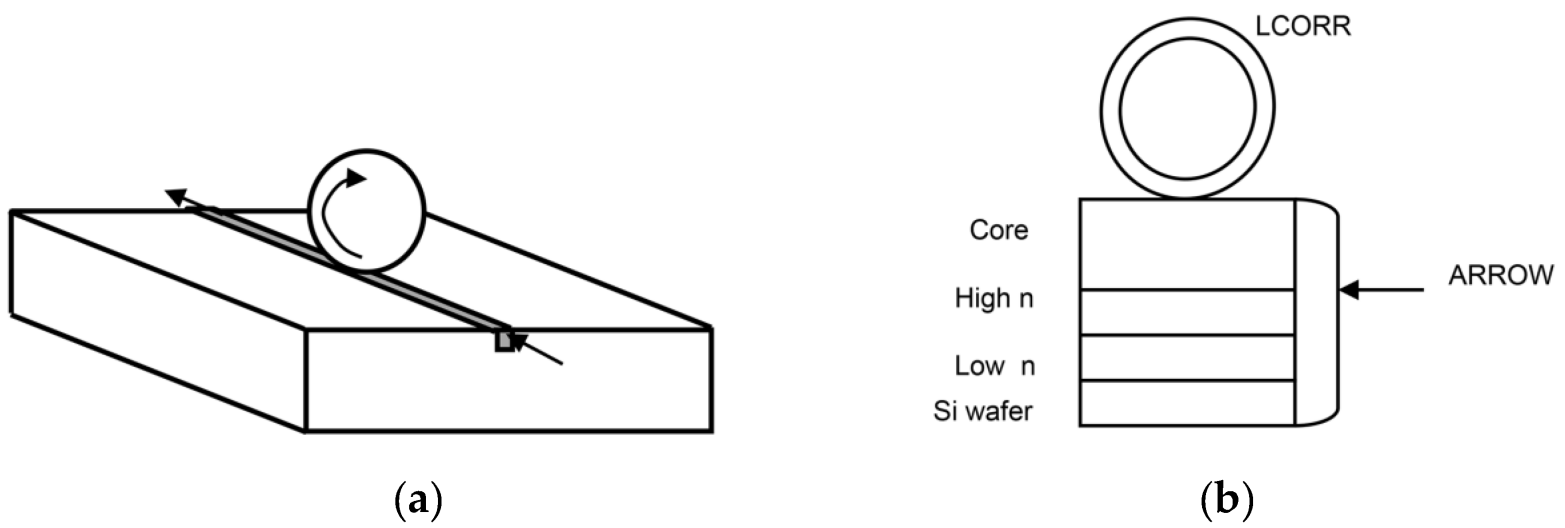
Sketch of hybrid WGM sensors: (**a**) microsphere coupled with a waveguide; (**b**) LCORR-anti-resonant reflecting optical waveguides (ARROW) system and cross-section viewed from the LCORR (liquid core optical ring resonator) on top of an ARROW.

Hybrid WGM systems are 3D or 2D structures like microspheres and microcapillaries with integrated couplers, e.g., optical waveguides [43], or integrated toroids coupled by fiber tapers [35]. Fan *et al.* developed a sensing architecture that combined anti-resonant reflecting optical waveguides (ARROW) and a 2D WGM sensor, based on a liquid core optical ring resonator (LCORR) [39]. The ARROW was brought into contact perpendicularly to the LCORR, thus coupling the light to and from the ring cross-section of the capillary (see Figure 5). A similar architecture was used to couple the light in a strip-line pedestal ARROW to a microsphere [44].

Non-integrated systems or fully-micro-optical systems are constituted by 3D or 2D structures, like microspheres, microcapillaries, microbottles and microbubbles, where the coupler is either a prism [45] or a tapered fiber [42]. Prism-based coupling adapts to any material, since the proper prism refractive index can be quite easily selected, and it is routinely used for robust coupling to high-*Q* millimeter-size crystalline disks.

**Figure 6:**
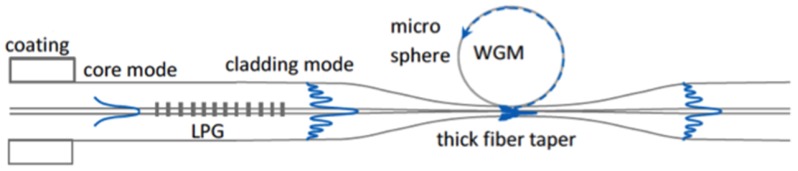
Sketch of a long period fiber grating (LPG) exciting the cladding mode followed by a ‘thick’ taper where coupling with the resonator takes place.

A silica fiber biconical taper is an excellent and easy-to-align coupling tool that allows fine-tuning of the fiber mode propagation constant, which depends on the taper thickness. The appropriate taper waist can be as small as 1 μm in diameter, with the fundamental mode extending significantly into the free space surrounding the taper; it offers great flexibility for an ideal phase and mode matching to the resonator modes. Its only disadvantage is being inherently fragile because of the thinness of the tapered region. In order to overcome this criticality, Farnesi *et al.* demonstrated a new method for coupling light, based on a long period fiber grating (LPG) followed by an adiabatically-tapered section of the fiber, which is about one order of magnitude thicker than the standard tapered fibers and, therefore, much more robust [46]. An LPG is characterized by a series of periodic refractive index changes in the core of a single-mode optical fiber. As is well known, a standard LPG has a typical grating period Λ in the range of 100–500 μm, and the coupling happens between the fundamental core mode and co-propagating azimuthally-symmetric cladding modes (see Figure 6). One or more attenuation bands characterize the transmission spectrum with the minimum of each band representing the coupling with a selective cladding mode [46]. In our system, the LPG is followed by a taper of the proper size, so that the excited cladding mode is close to the cut-off and strongly evanescent. Calculated minimum taper diameters are above 15 μm, depending on the excited mode and the surrounding medium. An all-in-fiber coupling system for quasi distributed and wavelength selective addressing of WGMRs along the same fiber was also proposed, which consists of replicating more times the same structure, namely a pair of identical LPGs with a fiber taper in between. The presence of the second LPG allows coupling of the light back into the core, so that all of the original information that has not yet been processed keeps propagating into the core mode and is transmitted till the end of the fiber, where it is collected by a single photodetector. Because of the wavelength selectivity of the pairs of LPGs, this method allows multiple selective coupling and interrogation (or selective addressing) of spatially-distributed micro-resonators [47].

A more detailed overview and analysis of the pros and cons of the different coupling systems is given in our previous papers [18,20,42].

## 3. Microspherical Resonators

Among the various geometries of WGMRs we have already mentioned, the microspherical one probably is the most investigated, due to the ease of fabrication in the laboratory, as well as in an industrial environment, from a large variety of materials, organic and inorganic. Liquid microspheres or microdroplets, which can be observed in nature, have indeed been the first WGMRs that were the subject of investigation. Mie, in his 1908 paper, provided a complete analytical solution of Maxwell’s equation for the scattering of electromagnetic radiation by spherical particles; thus, the electromagnetic field inside and outside of a particle could be represented by a series of functions that are special solutions of the Maxwell equations [48]. Almost 70 years later, while experimentally checking the Mie–Debye theory, Ashkin and Dziedzic observed, in silicone oil droplets (4–30 μm in diameter), a series of sharp optical resonances, which were attributed to dielectric surface waves [49]. Shortly thereafter, the WGM resonances were observed in individual dye-impregnated polystyrene microspheres (9.92 μm in diameter) suspended in water and illuminated by an argon laser at 457.9 nm [50]; the proof of lasing action in individual ethanol droplets (60 μm in diameter) containing Rhodamine 6G, pumped by a cw laser at 514.5 nm [51], was then opening the way to practical applications of the WGMRs. Let us now summarize here the fabrication methods of bulk polymer, glass and crystal microspheres, as well as of hollow glass microspheres, also called microbubbles.

### 3.1. Polymer Microspheres

Optical polymeric materials have found wide application in the area of optical components and photonic circuits, thanks to the possibility of tailoring their properties, which may include, besides high transparency, large electro-optic coefficients, relatively large thermo-optic coefficients and photorefractivity. Extremely transparent and reliable passive optical polymers have been made commercially available, while research advances have permitted the synthesis of excellent active polymers. Several papers have been published on polymeric optical passive and active components, including low-loss waveguides, high-speed electro-optic modulators, thermo-optic switches, light-emitting elements, broadband solar cells, flexible displays, and so on [52]. Resonant structures, like optical microring resonators (OMR), which are characterized by strong light confinement in a small modal volume, a high *Q*-factor and a narrow resonant bandwidth, have been extensively exploited in various applications; as a recent example, an all-polymer OMR was designed for specific heavy metal (hexavalent chromium) sensing [53]. As a further prospective, we can mention that novel polymeric materials are being synthesized that are suitable for interaction by assembling with DNA in order to obtain new biomaterials with interesting nonlinear optical properties [54]. It has to be stressed, indeed, that most of the applications of polymeric microspheres are in the field of biomedicine: microsphere technology, in fact, serves as an efficient and effective platform for cell applications (*in vitro* cell culture and *in vivo* cell delivery) due to its mimicry of the 3D native environment, high surface area/volume ratio and ability to isolate the entrapped cells from the environment [55]. On the optics side, we can remark that polymer chemistry allows us to prepare truly monodisperse microspheres, *i.e.*, having a monodispersity (ratio of weight-average diameter to number-average diameter) less than 1.005, either by physical methods, such as emulsification, coacervation and spray-drying, or by chemical methods, such as heterogeneous polymerization [56,57,58]. An interesting book on nanochemistry devotes a chapter to the synthesis of organic and inorganic microspheres, even if especially in view of the synthesis of photonic crystals [59].

One of the possible routes to fabricate polymer microspherical WGMRs follows the same procedure as for silica microspheres, *i.e.*, starting with the fabrication of a fiber (here, in PMMA) and then of a microsphere by melting the tip of the same fiber; the *Q*-factor of the microspheres prepared this way was found to be ≈5 × 10^4^, but can be increased up to 10^6^ when purifying the material and, instead of melting the fiber, using a microsyringe to deposit a controlled drop of the purified PMMA on the tip of the fiber [57]. Polydimethylsiloxane (PDMS) microspheres were also fabricated with a slight modification of the process described above [58]. By utilizing fiber tapers with different diameters and adjusting the viscosity of the pre-cured PDMS, microspheres with diameters ranging from 100 μm–1 mm were fabricated. Another approach involved the fabrication of dye-molecule-doped microspheres in a microfluidic channel [60]; starting from an UV-curable resin (NOA81, Norland, Cranbury, NJ, USA)mixed with 10^−2^ mol/L Rhodamine 6G (R6G) and 5% (volume ratio) of ethanol, microspheres with monodispersity ± 2.1% and size controllable in a range between 20 and 80 μm were produced. The optical quality of these R6G-doped WGMRs was poor (a *Q* ≈ 10^3^ was experimentally inferred, probably limited by the resolution power of the spectrometer used), but high enough to observe lasing in a microsphere of radius 73.87 μm at a threshold of about 16 μJ/mm^2^ [60]. Several other fabrication techniques were also used; for instance, monodisperse controlled micron-sized dye-labeled polystyrene particles have been prepared by two-stage dispersion copolymerization with polymerizable dyes [61] and used in a microspheres-microtube system to study coupled microspheres and to learn more about their fundamental properties [62]: two spheres with a diameter of 6 μm were placed into a microtube with an inner diameter of 6.2 μm and coupled in such way that the evanescent field of WGM resonances of each separate sphere coherently added up together, so that it was possible to experimentally demonstrate coherently-coupled photon states in such microsphere resonators. In polymeric materials, an intermediate step between photolithographed microrings and spherical resonators is represented by structures with a suspended cavity design, which can be produced by proton beam writing: an example is the microlaser made in a Rhodamine B (RhB)-doped SU-8 polymer [63].

For completeness, we can add that additional techniques of the synthesis of polymeric microspheres have been developed for biomedical applications, and cross-fertilization of these two application areas could give interesting results. Just to mention two examples, polystyrene micro- and nano-spheres with uniform dimensions and smooth surfaces were produced by electrospray [64], and very uniform spheres with average diameters from ≈5–500 μm were obtained by spraying a polymer-containing solution through a nozzle with (i) acoustic excitation to produce uniform droplets and (ii) an annular, non-solvent carrier stream allowing further control of the droplet size [65].

### 3.2. Glass Microspheres

Most microspherical WGMRs are made in glassy materials; pure silica represents the preferred choice, due to the available very high purity and nanometer-scale surface smoothness: these two characteristics made possible the achievement of a top value *Q* ≈ 8 × 10^9^, with a corresponding finesse *F* ≈ 2.2 × 10^6^ [66]. The observed independence of *Q* from wavelength, together with the data obtained by atomic force microscopy, suggests that it is not limited by material absorption, but possibly by residual surface inhomogeneities. Multi-component and non-oxide glasses are also of great interest, even if with higher absorption coefficients, because they can offer more potentialities for new functionalities and for mid-IR operation. In all cases two main techniques for the fabrication of microspherical resonators can be adopted, one based on melting processes, the other one on sol-gel chemistry; they will be briefly described in the following.

#### 3.2.1. Melting of Glass Microspheres

Perfect micrometer-sized glass spheres are known to be formed by surface tension, and that makes melt cooling a very popular method for fabricating microspheres. A few options can be devised to produce a number of microspheres at once, in a certain range of sizes; in some cases, one starts from raw glass components, melting them and then dropping the viscous glass onto a spinning plate [67] or pouring it in a fine stream into liquid nitrogen [68]. Another approach is to synthesize a given glass, to grind it into micron-sized particles and then to drop them through a microwave plasma torch [69,70] or a vertical tube furnace [71]; the plasma torch is suitable for silica glass, as well, while the designed furnace is functional only with glasses having a relatively low glass transition temperature, such as heavy metal fluoride glasses, chalcogenide glasses and phosphate glasses. An experimental device for obtaining glass microspheres in a rotating electrical arc was developed, as well, transforming the glass powder with arbitrarily-shaped particles into microspheres with diameters between 2 and 24 μm [72]. In all of these systems, the re-melted glass grains acquire a spherical shape by surface tension before being hardened by the air flow and falling into a proper container; not all of the grains, however, are effectively assuming a perfect spherical form.

The above techniques have pros (a large number of microspheres is generated at once, and even continuous production can be conceived) and cons (The spheres have a rather large size distribution and are difficult to manipulate. It is necessary to place them under an optical microscope and to sort them by size, while also checking their surface quality. The selected spheres may be picked up with a glass capillary connected to a vacuum pump and, then, one at a time, glued to a fiber end face.).

Single spheres with the desired diameter and reproducible high quality are produced by melting the tip of a glass fiber; this common method is very inexpensive and allows one a very good control of the microsphere diameter. Upon heating the distal tip of a fiber, e.g., a standard telecommunications silica fiber, or of any small glass rod, the glass reflows to form a spherical volume under the influence of surface tension. Due to the high viscosity of glass, the reflowed structure becomes highly spherical (eccentricities of the order of 1%–2%) and extremely uniform. The spherical surface has very low intrinsic roughness (measured RMS roughness of the order of 1 nm or lower) and thus presents very small surface scattering losses. Different research groups have used different heating sources to induce the reflow process, such as an oxygen/butane or nitrous oxide/butane microtorch [73], a high-power (e.g., a CO_2_) laser [58,74] or an electric arc [75] as in a commercial fiber splicer [76,77]. It can be noted that accurate experimental works were made to produce and measure integral microspherical fiber tips for the application as sensing probes [78,79]. For the details, we direct the interested readers to the given references. This group of techniques allows one to produce microspheres with diameters usually in the range *d* to 2*d*, where *d* is the outer diameter of the uncoated fiber or rod; smaller spheres, say from 20–100 μm in the case of conventional telecom fibers, may be obtained by first tapering the fiber in order to reduce the diameter of the tip. The minimum diameter we obtained by using the fiber splicer on tapered fibers was about 40 μm [76].

#### 3.2.2. Sol-Gel Glass Microspheres

Sol-gel chemistry is attracting a growing interest as a very flexible and inexpensive method for producing optical materials. The possibility of starting from molecular precursors and elementary building blocks permits one to tailor structures at the molecular level and to create new materials with enhanced performances; sol-gel may be used to produce either bulk microspheres or to coat a spherical particle, thus adding novel properties (or even substituting the original material properties).

Many protocols can be used to realize sol-gel silica spheres: monodisperse silica nanoparticles, in particular, can easily be synthesized via the base-catalyzed hydrolysis of tetraethyl orthosilicate (TEOS) [80]; the resulting silica spheres may have a diameter in the range from 150 nm–2 μm, with a typical size dispersion around 3%. This method cannot be used if one wants to incorporate rare earths into the glass, because lanthanide salts form insoluble lanthanide hydroxides in basic environments. The problem, however, may be overcome by using acid catalysts [81,82]: In this case, one obtains silica microspheres with different diameters, ranging from a hundred nanometers to ≈ 150 μm, always with a very high surface quality. The synthesis of high-purity dense silica microspheres from organic and inorganic acid hydrolysis of TEOS has been demonstrated using a variety of acid and water mixtures.

Sol-gel also can be considered the best among the various techniques used to deposit coating layers onto spherical microresonators in order to tailor or optimize their optical properties. Most of the work in this field concerned methods that lead to uniform coatings, which can guarantee preserving the high *Q* of the microresonator. Not always is uniformity guaranteed: This is the case of silicon nanocrystal films deposited by electron-beam evaporation onto fused silica microspheres in order to modulate the fluorescence spectrum [83] and of air-sprayed films of a silicone oligomer onto glass spheres placed onto a Teflon sheet [84]. In the latter case, the spheres were coated uniformly, except one side, which was attached to the Teflon sheet surface and remained flat (hence the name terrace-microspheres) [84].

The coating may have a lower or higher refractive index than the microsphere itself, and its function changes accordingly. A lower index coating makes the microsphere insensitive to the surrounding environmental conditions and ensures the stability of the whispering gallery modes; it has the disadvantage that even the coupling via evanescent waves becomes more difficult. On the contrary, a higher index coating is “attracting” the propagating waves, and the electrical field of the WGMs is almost entirely inside the coating, unless it is very thin. Given a generic core-shell structure, the WGMs may be described by the Aden–Kerker generalization of the Lorentz–Mie scattering theory; resonances of a layered microsphere (diameter of ≈3 μm) were analyzed by Hightower and Richardson [85]. The WGMs of such a core-shell structure can have superior characteristics compared to simple uncoated microspheres, in particular as far as the thermal stability of the WGM modes is concerned.

The coating, moreover, can improve the microcavity properties and/or add novel optical functions [86,87]; the only limitation may be due to the fact that the *Q* of a microsphere coated with an optically-active material is generally reduced compared to the *Q* of the uncoated one, due to the absorption in the coating and to the scattering at the sphere-coating interface. Spherical microresonators have been coated by a number of materials, such as, just to mention a few of them, conjugated polymers [88], PMMA [89], fluorescent dyes [90,91,92], nanocrystalline silicon [83], silica-hafnia glass and glass-ceramic [20,93,94], polystyrene and calcium fluoride [95], zeolite [96], HgTe quantum dots [97], etcetera. The low-pressure chemical vapor deposition (LPCVD) method was found to produce films of very low surface roughness, an important requirement for the films to be usable on WGMRs [98]. Recently, thin LPCVD silicon-rich oxide films (SiO_x_, 0 < x < 2), which lead to nanocrystalline-Si films by thermal annealing, were successfully produced with a very smooth surface (r.m.s.vertical surface roughness less then 3 nm, with lateral surface structures below 50 nm).

The sol-gel technique is effectively exploited especially for depositing active coatings on microspherical resonators due to the fact that it allows for precise control of rare earth ion concentration and high solubility, as well as easy control of the refractive index of the coating [75,94,99,100]. Another very important field concerns the deposition of coatings for the functionalization of microspherical resonators to be used as biosensors; this subject will be treated in the following section (Section 4) of this paper.

#### 3.2.3. Crystalline Microspheres

Optical-quality single-crystal spheres can easily be manufactured by hand lapping and polishing techniques; several types of crystalline (quartz, sapphire, YIG) spherical resonators with mm size have been effectively used as resonators at microwave frequencies [101,102,103]. Single-crystal spherical YIG resonators with a diameter in the range of 200–1000 μm have also been produced, which support resonant frequencies of electromagnetic modes in the sphere above 100 GHz [103]. BaTiO_3_ crystalline spheres from 3–7 mm in diameter were also manufactured by polishing and tested as optical resonators at 532 nm [104]. A pulsed laser irradiation method was invented to selectively heat colloidal nanoparticles and, thus, synthesize size-tailored semiconductor and metal submicrometer spheres, with a narrow distribution of sizes [105]. However, fabrication of single crystalline particles with high sphericity and micron sizes remains a challenge, due to the fact that faceted surfaces normally appear, reflecting the atomic arrangements. A recent work showed that the fabrication of single-crystal semiconductor microspheres that have surfaces with atomic-level smoothness is possible by performing laser ablation in superfluid helium to create and moderately cool a melt of the anisotropic semiconductor material [106]. Fabrication of CdSe (wurtzite structure), ZnSe (zinc blende structure) and CeO_2_ (cubic structure) single-crystalline microspheres, with diameters in the range from several tens of nanometers to around 2 μm, was demonstrated; their crystalline structure was confirmed by high-resolution TEM and electron diffraction patterns [106].

### 3.3. Microbubbles

Another resonating structure with spherical symmetry that was proposed a few years ago is the microbubble, potentially very useful for microfluidic bio- and chemo-sensors [41]. Microbubbles can be blown by pressurizing a capillary while softening it by using CO_2_ laser heating [41] or an electric arc discharge [107]. In the latter case, the heat was uniformly distributed around the midsection of the microcapillary by arc discharge and simultaneous the rotation of the U shaped holder around the capillary. Reproducibility is ensured by the control of all physical parameters of the process (duration and power of arc discharges, gas pressure, rotation speed, distance between capillary and electrodes). *Q* factors up to 10^7^ both at 773 nm and 1550 nm were routinely achieved [108]. The sensing capability of microbubble resonators is related to the intensity of the electromagnetic field inside the hollow cavity and, therefore, to its wall thickness, which typically should be lower than 2 μm; hence, it is extremely important to measure the thickness to assess the effective capabilities and sensitivity of the sensing device. A non-destructive method for measuring the shell thickness of a microbubble using reflectance confocal microscopy was developed and successfully tested [109].

## 4. Surface Functionalization

The surface functionalization or chemical modification of the transducer’s surface is of critical importance for producing effective biosensors, which have to be very selective. Proteins, for instance, adhere to any glass surface, thus increasing the unwanted non-specific effects. The coating layer, however, is crucial and has to be very thin, in the range 10–100 nm (below the evanescent penetration depth) and homogeneous in order to preserve the high quality of the transducer [19,110].

### 4.1. Selection of the Biorecognition Element

The ultimate sensitivity and specificity of the sensor is given by the biorecognition element (BRE), irrespective of the transducer’s sensitivity. The choice of the BRE should then take into account several parameters, such as high specificity, high affinity and stability. The most commonly-used BREs are antibodies, antibody fragments, complementary DNA and aptamers and, in some cases, streptavidin and enzymes, as well. Antibody-based sensors are also called immunosensors, while aptamer-based sensors are called aptasensors. The antibody shows high affinity and specificity towards the antigen, due to their molecular complementarities. Complementary DNA has also a high specificity and affinity. Aptamers are functional molecules selected *in vitro*; they have high specificity and affinity, and even small changes in the target analyte may disrupt the binding; they can in principle be selected *in vitro* for any given target, ranging from small molecules to large proteins and even cells, thus making it possible to develop a wide range of aptasensors. Enzymes are specific in both the reaction they catalyze and the substrate they recognize and are subject to the regulation of their activity by other molecules. Therefore, the use of enzymes can be advantageous owing to enzyme specificity and to the amplification phenomena given by enzyme catalysis. A major disadvantage is the dependence of the enzyme activity on physical and chemical environments. Streptavidin is highly specific to biotin/biotinylated proteins, but usually is based on physisorption, a non-covalent method that provides a simple method for proof-of-concept tests, but it has severe limitations for complex assays that require reproducible surface blocking and surface regeneration [45,111,112]. Careful attention must also be paid to factors, such as molecular orientation, specific attachment site and spatial control, in order to avoid cross-reactivity in multiplexed assays.

### 4.2. Covalent Functionalization Techniques

**Figure 7:**
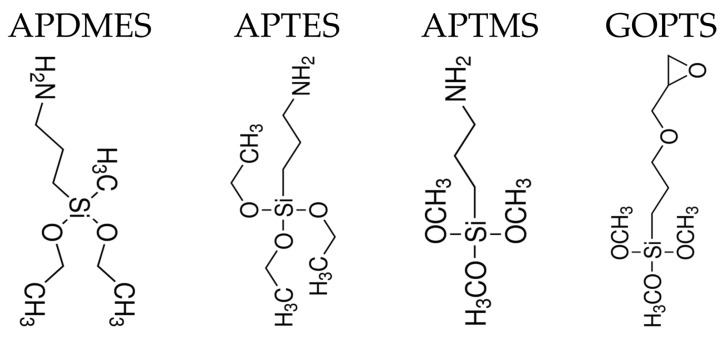
Chemical structures and abbreviations of the silanes mentioned in this review.

There are several ways of functionalizing the surface of a biosensor, and covalent binding provides the best stability and durability. Among the various approaches, the most common ones are based on the silanization of the glass or silicon surface through covalent binding of the silane groups with the glass surface. The approach shows a reduced non-specificity and enables a further functionalization with ligands or receptors. Silane deposition can be made following wet or dry deposition protocols; there are a variety of solvents that can be used for silane deposition, such as ethanol, dimethylformamide, isopropanol, toluene or acetone, through fluid, vapor phase or spotting methods. The literature reports a variety of silanes that have been effectively used [113,114,115,116]. In order to provide amine functionality, aminosilanes have been extensively used, even though aminoalkoxysilanes are highly reactive towards water, which can cause uncontrolled polymerization/oligomerization of aminosilanes in solution. The most used aminosilanes in the literature are (3-aminopropyl)triethoxysilane (APTES), (3-aminopropyl)trimethoxysilane (APTMS) [113] or 3-aminopropyldimethylethoxysilane (APDMES) [117]. APTES is more commonly used because of its lower cost; it has three ethoxy groups per molecule and is capable of polymerizing in the presence of water, which can give rise to a number of possible surface structures: covalent attachment, two-dimensional self-assembly (horizontal polymerization) and multilayers (vertical polymerization). Vapor phase silanization has also been reported to produce smooth APTES monolayers. Due to the presence of a single ethoxy moiety in each APDMES molecule, its reaction with silica is easier to control and should result in amine-functionalized monolayers. Other silanes have been used to a lesser extent, for instance epoxy-silanes, such as 3-glycidoxypropyl-trimethoxysilane (GOPTS or GPTMS) [28,118], or mercaptosilanes, such as 3-mercaptopropyltrimethoxysilane (MPTMS) [119]. The former creates epoxide surfaces, while the latter creates thiolated surfaces. Figure 7 shows the chemical structures of the mentioned silanes.

**Figure 8:**
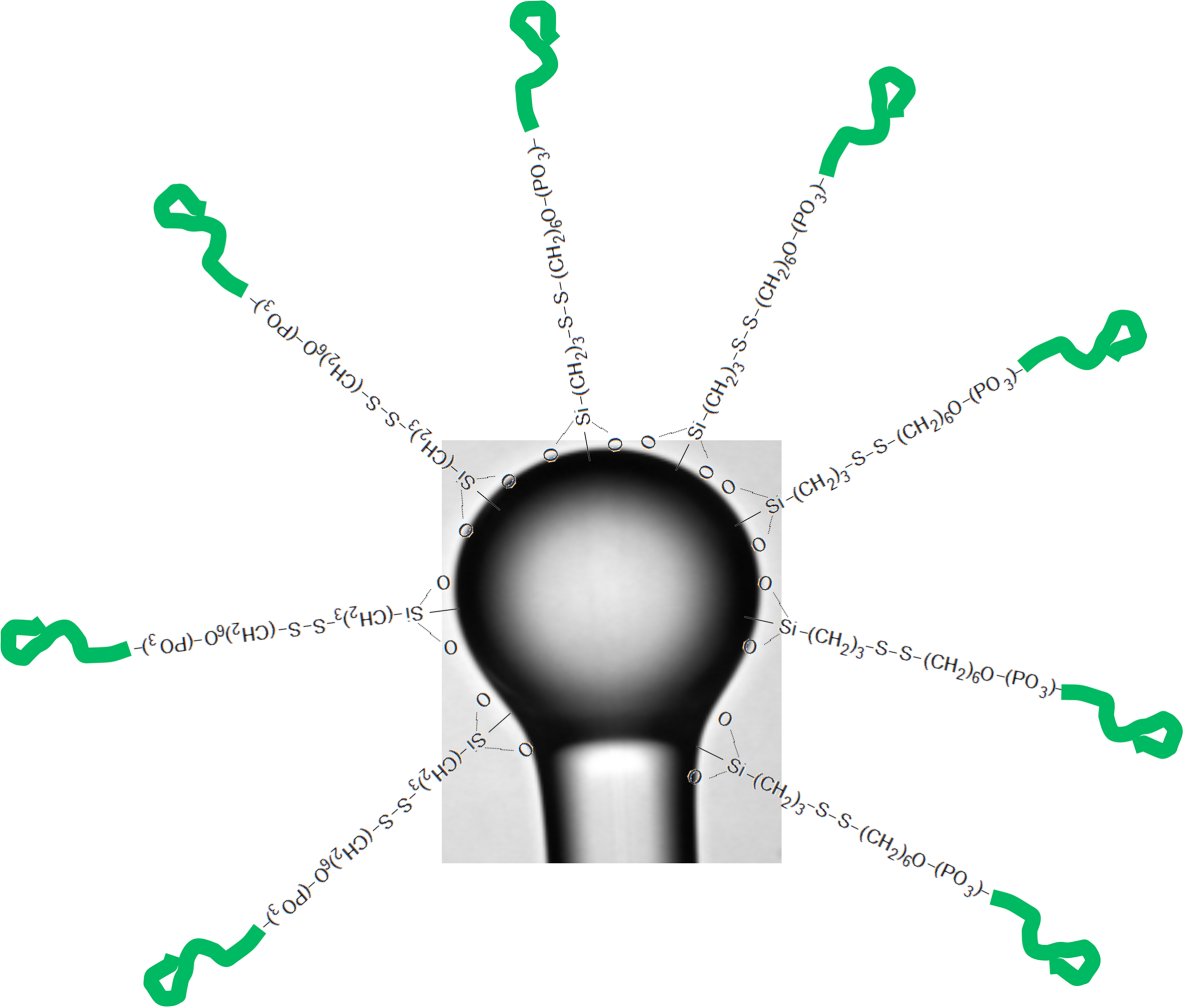
Functionalization of a WGMR for an aptasensor: after activation of the surface with piranha solution, silanization with 3-mercaptopropyltrimethoxysilane (MPTMS); and thiol bonding to the thrombin aptamer.

As already mentioned, silanization is a disordered process that can produce multilayers, and special care is needed in order to preserve the *Q* factor during all covalent functionalization procedures. After silane functionalization, the surface can be further modified for attachment to the recognition element of interest. This bioconjugation protocol is usually based on cross-linking chemistry. Among cross-linkers, we can find glutaraldehyde [120,121], hydrazino-nicotinamide (HyNic)/4-formylbenzamide (4FB) [122] or 1-ethyl-3-[3-dimethylaminopropyl] carbodiimide hydrochloride (EDC) and N-hydroxysuccinimide (NHS) [110]. The cross-linkers help to bind the amines to the surface of the device with the amine/carboxyl groups on the BRE. Figure 8 shows an example of spherical WGMR functionalization with MPTMS and thrombin aptamers.

Very recently, an attractive functionalization procedure based on a photochemical reaction has been tested in hollow capillaries [123] and polymeric goblets [124]. UV-light exposure is used to selectively activate the BRE capture. Up to now, the sulfo-NHS-LC-diazirine cross-linker has been used in hollow WGMRs [117], while the benzophenone-dPEG3-biotin cross-linker has been used for the goblets [29]. Such an approach opens up the road for efficient multiplexed detection. In some cases, an orienting layer is also used in order to avoid random coverage of BRE into the silanised surface; for instance, in order to avoid random orientation of the antibodies, an orienting layer of protein G (an immunoglobulin-binding protein expressed in groups C and G Streptococcal bacteria) may be immobilized covalently, which binds antibodies with a high affinity [118].

The last step is constituted by the passivation of the active sites in order to minimize the non-specific adsorption that might give rise to false positives. Passivation is achieved through the so-called nonfouling surface elements or blocker elements; poly(ethylene glycol) (PEG) [125], zwitterionic polymeric brushes [126] or b-mercapto-ethanol (MP-ET) [119] have been successfully used as blocking elements. For some applications, re-usability is desirable, in order to use the same functionalized surface in consecutive assays. It can be a partial regeneration, by removing only the BRE (inter-assay regeneration) or a total sensor regeneration. In the case of WGM aptasensors, two different chemicals have been used successfully for regeneration: NaOH solution [119] or a pH = 2 glycine-HCl solution [28], that is also useful for the disruption of protein-protein interactions [122]. Total device regeneration can be done by using hot piranha (3:1 H_2_SO_4_-H_2_O_2_) solutions [127] or dilute hydrofluoric acid (HF) solutions [128].

## 5. Biosensing by WGM-MSR: State of the Art

The application area where WGMRs have better proven their unique properties is that of sensing; indeed, in very high *Q* resonators, the trapped photons are able to circulate on their orbits several thousand times before exiting the WGMR by the loss mechanism, and this very long interaction path makes possible the detection of very small external perturbations, such as those induced by the presence of a nanoparticle [129]. An interesting analysis of the dependence of sensitivity on microsphere size is presented in [130]; by using a 9-μm diameter tellurite sphere, refractometric sensing with a sensitivity of 7.7 nm/RIUwas demonstrated, and according to the theoretical calculations, it was concluded that these high index microspheres are suited for the detection of nanoparticles smaller than 50 nm, while silica spheres are more suitable for the detection of the uniform change of the exterior refractive index and for larger nanoparticles. Despite the relatively low *Q* factor, a 9-μm diameter pure tellurite sphere should be capable of detecting a nanoparticle of 30 nm in diameter, and a 5-μm micron diameter sphere should detect a 10-nm diameter nanoparticle [130].

Going to the specific area of biological sensing, the main technical requirements, as already mentioned, concern selectivity, sensitivity, stability and reversibility. These are mostly provided by the biochemical receptor (antibody, antigen, DNA, enzyme, aptamer), the sensitivity being also provided by the quality of the optical platform; in the previous section, the importance of the functionalization of the WGMR surface has been underlined. Other important characteristics are high signal-to-noise ratio (SNR), short response time, low limit of detection (LOD), integration capabilities and high sensitivity at low cost in real samples. Thermal fluctuations may represent a critical issue, as they can induce undesirable thermo-optic and thermo-mechanic effects, so providing a noise source; a theoretical analysis is presented in [131], together with the results of the measurements of thermo-refractive noise in microspheres. Even though some authors claimed that the thermo-optic effect can greatly enhance the LOD, permitting going down to single molecules, it has been proven that thermo-optic effects are far too small for enhancing the LOD [132].

A self-referenced WGM biosensor, proposed in order to reduce noise (thermal, non-specific binding, *etc.*), was based on so-called photonic molecules, namely coupled microspheres [133]. Temperature compensation of optical microresonators may also be achieved by using a surface layer with a negative thermo-optic coefficient [95]. A more recent approach that permitted enhanced detection of single-nanoparticle exploited the mode splitting in a silica microtoroid WGMR via Raman gain-assisted loss compensation and a WGM Raman microlaser; the mode splitting provides a self-referencing scheme immune to laser frequency noise and the thermal drift of resonances [134].

**Figure 9:**
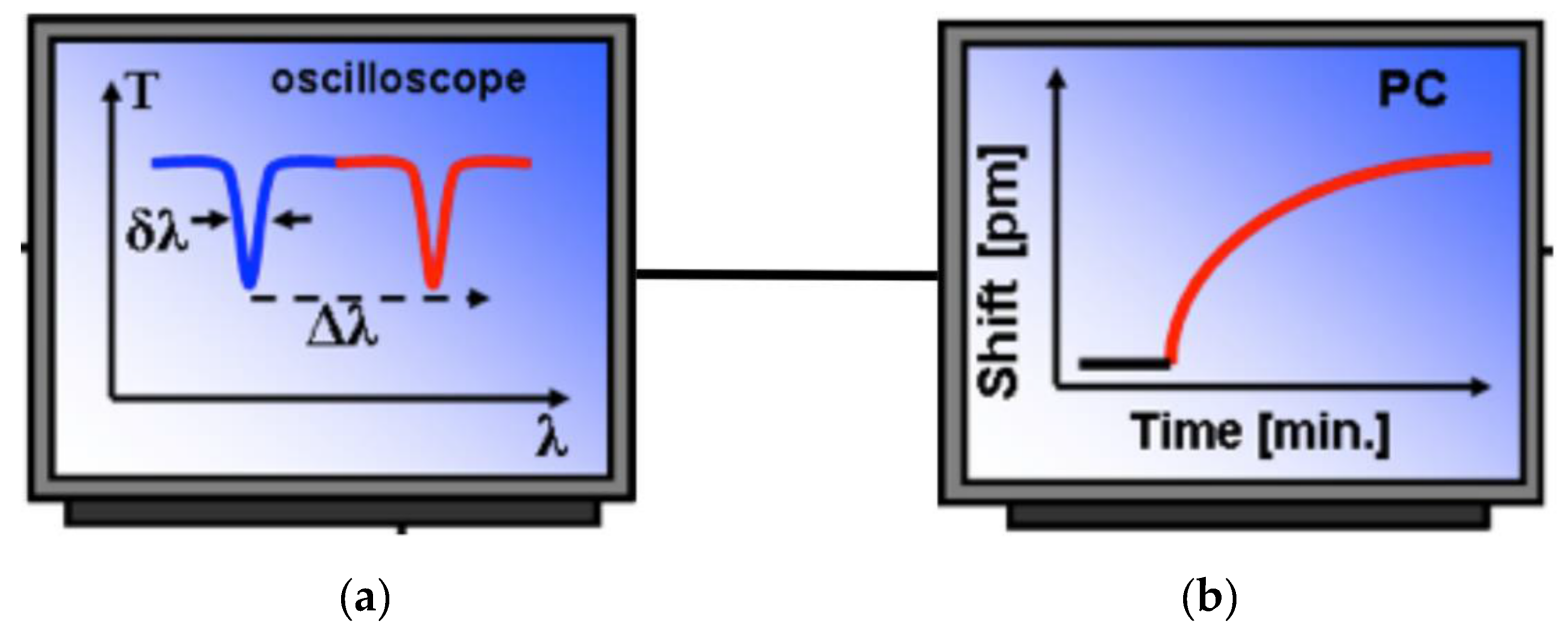
(**a**) Schematic WGMR transmission line shape before (blue) and after (red) a resonant shift; (**b**) Resonant shift versus time (sensorgram).

The most common detection scheme in WGM sensors uses a narrow linewidth laser, which can be finely swept at very low frequency around a resonance by a few GHz: in this way, resonant wavelengths can be selectively excited and detected. The light transmitted through the coupler-WGMR system can be then monitored at the output of the biconical tapered fiber using a detector connected to an oscilloscope. Figure 9 shows schematically the working principle of a WGMR sensor.

A method that was claimed to have excellent detection capabilities was based on a microlaser detection scheme [135]. As the ultimate detection limit is set by the laser linewidth, which can be as narrow as a few Hertz for this kind of device and certainly much narrower than the resonance linewidth of any passive resonator, a WGM microlaser sensor, regardless of whether it utilizes reactive shift or mode splitting, has the potential to detect smaller objects beyond the reach of passive resonator sensors.

Very recently, an alternative measurement approach has been demonstrated, based on the so-called ringing phenomenon [136]; by this term, one refers to the oscillations, which can be seen in the transmission spectrum when a tunable cw laser sweeps over a high *Q* whispering-gallery mode with the help of a fiber taper in a fast speed [137]. The ringing phenomenon had been previously used to measure the characteristics of the modes, such as the *Q*factors and mode-coupling strengths [138], but here, it was proven that it can be exploited to sense environmental changes; Experiments were carried out with silica microspheres with diameters of ≈112 μm and ≈52 μm, and it was estimated that polystyrene particles of an ≈70 nm radius should be detected [136].

**Figure 10:**
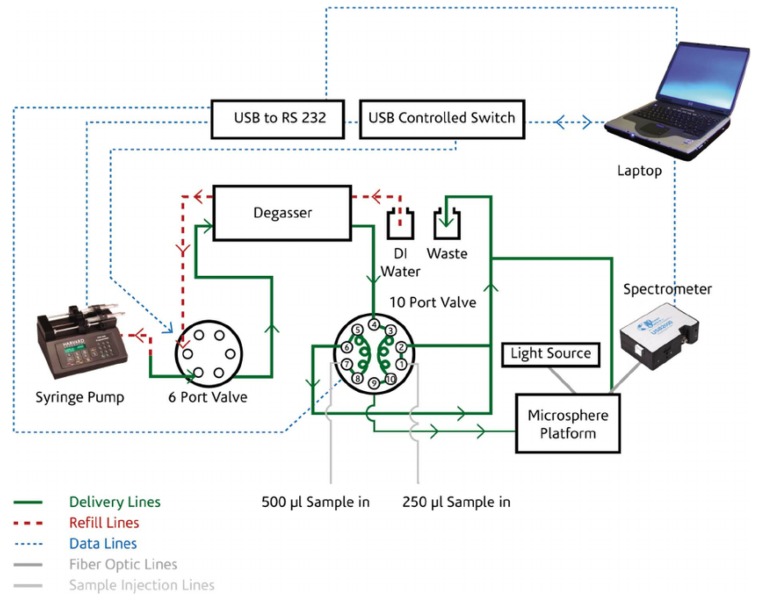
An experimental setup for WGMR biosensing, which includes the controls of the flow of water and analyte-containing solutions past the microsphere. Reprinted with permission from [141].

Sample delivery is also a critical step in a biosensor device. Biochemical samples typically are in the form of aqueous solution; microfluidics is one of the most commonly-used methods of sample delivery. Ideally, the fluidics design should take into account sample injection and drainage, the reduction of sample volume and detection time. A robust package of a microsphere and a tapered fiber is a quite challenging task, even in a dry environment; in air, however, some solutions have been found, such as the one achieved by using a glass tube, two glass plates and UV glue, which allows one to keep the *Q* factor of the microsphere as high as 1.08 × 10^8^ and to make the transmission spectrum more stable and insensitive to the external vibration and air flow [139]. Several attempts to integrate the microsphere-taper system and the fluidics have been made, as well, e.g., by immersing the microsphere in a fluidic cell whose bottom window is constituted by the coupling system itself, e.g., a fiber prism (fiber tip polished at 74 degrees) [140] or the flat side of a hemispherical prism [141]. Several tests of WGM microspherical biosensors were carried out with the sphere immersed in a finite volume (closed cell) or even in a drop of the solution containing the analyte, but in these cases, depletion of the ligand reservoir may become significant, and the binding may rapidly saturate [112]; for real applications, a fluidic system is necessary. Figure 10 shows an experimental setup where pumps and valves are used to control the flow of water, alcohol and analyte-containing solutions through the microfluidic chip and past the microsphere. The fluidic system, which includes a syringe pump, a six-port valve and a ten-port valve, is controlled by custom software, which also receives the output from the spectrometer and plots the transmitted light as a function of time [141].

**Figure 11:**
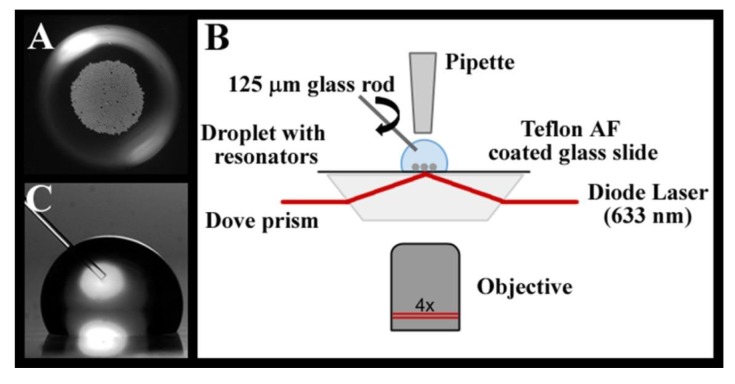
(**A**) Top view of some 500 barium-titanate (38 μm in diameter) microspheres immersed in a 10-μmL PBS droplet. (**B**) Sketch of the WGM droplet assay. The light from the excited resonators is imaged from below (4× objective) and detected by an APD. (**C**) In active mixing mode, a 125-μm diameter glass rod is rotated at 5000 rpm in the PBS droplet. Reprinted with permission from [142].

Another factor to be considered concerns the velocity of analyte diffusion, especially in unstirred solutions; for species at low concentration, diffusion to the sensing element often represents a limiting factor in the time required to carry out analysis [142]. Small volume (10 μL) droplet assays utilizing WGM spherical resonators with a 38-μm diameter have been demonstrated as a promising label-free sensing platform; active mixing of the small droplets using a small, spinning glass rod results in rapid mass transfer and reduces sensor response times to seconds, which enables nanomolar protein quantitation using initial binding rates within the first minute following injection. Figure 11 shows a sketch of the experimental setup, where a Teflon-coated glass slide with a PBS droplet is placed onto a Dove prism coated with high index immersion oil; the prism is used to excite whispering gallery modes in the barium-titanate microspheres immersed in the droplet [142].

**Figure 12:**
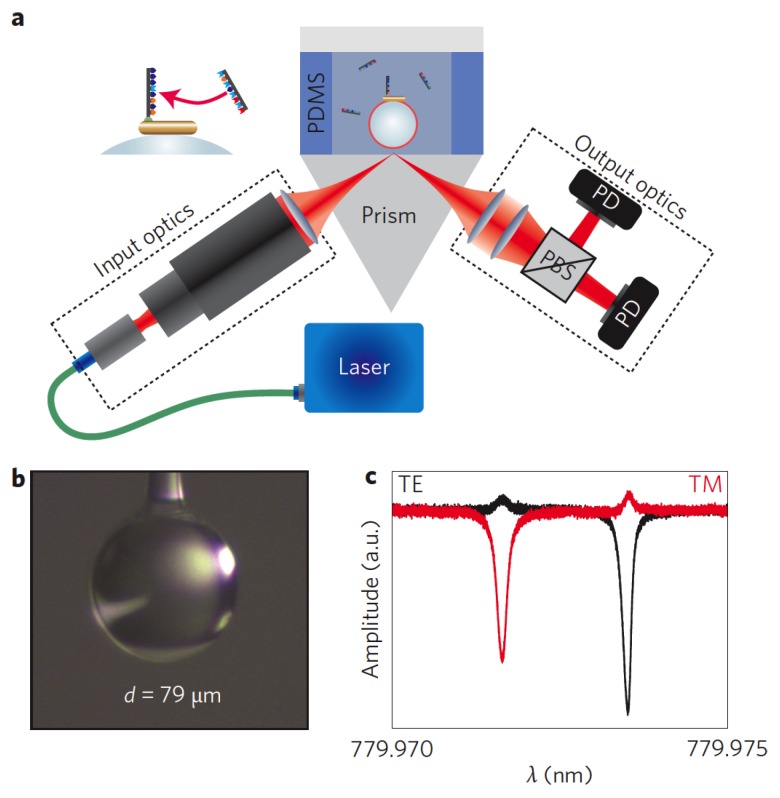
Experimental setup of the WGM plasmon-enhanced biosensing platform. (**a**) A prism coupler is used to excite WGMs in a glass microsphere. The liquid sample cell is made in PDMS. The inset shows a plasmonic nanorod enabling detection of single oligonucleotides. (**b**) Photo of the spherical WGMR, with a diameter in the range of 60–100 μm. (**c**) Example spectra for TE(black) and TM(red) polarization obtained with an ≈79 μm microsphere in water, exhibiting typical experimental *Q* values around 5 × 10^6^. Reprinted with permission from [146].

The first tests to demonstrate the suitability of WGM spherical biosensors were performed in 2002–2003 [111,114]. Since then, several other demonstrations have followed, and the interested reader is referred to some recent review papers for a comprehensive list [19,21,143]. Just to mention a few of the most significant results, we can remind the reader that even single molecules and nanometric biological objects have been detected, such as the Influenza A virus [135,144], an RNA virus known as MS2 [17], proteins such as interleukin-2 [145] and streptavidin [132,145], single 8-mer oligonucleotides [146], exosomes, ribosomes, mouse immunoglobulin G and human interleukin-2 [147]. Thermo-optic and reactive mechanisms for label-free sensing of bio-particles have been compared theoretically for WGM resonators (sphere, toroid) formed from silica and stimulated into a first order equatorial mode [132]. The detection of single oligonucleotides, as reported in [146], can only be achieved by significantly increasing the biosensor signals; a possibility for such enhancement is given by plasmon resonance: a plasmonic nanoparticle can enhance the optical field strength at the microcavity surface, amplifying the resonance wavelength shift upon binding of an oligonucleotide [146]. A microcavity biosensor platform (see Figure 12) was developed, based on prism coupling and exhibiting improved mechanical stability due to the elimination of coupling fiber fluctuations. Since evanescent light coupling is restricted to the laser focal spot, the susceptibility to nanoparticle fouling at the prism surface is greatly reduced. The prism, moreover, can be easily cleaned and repeatedly integrated with different polydimethylsiloxane (PDMS) liquid sample cells.

A proposal for an efficient integration of the fluidics with a WGMR was suggesting the use of hollow cylindrical microstructures, like microcapillaries or liquid core optical ring resonators (LCORRs): they exhibit lower *Q* factors (≈10^6^) than spherical WGMRs, but have the advantage that the fluidics is part of the optical structure itself, so that efficient and rapid delivery of analytes in liquid and in air is guaranteed [148]. On the other hand, the geometry of the capillary can be changed to create microbubbles, which strongly confine the WGM in the axial direction as well, and therefore, significantly reduce the mode volume; microbubbles utilize their interior surfaces to capture the analyte and, by suitably decreasing the wall thickness, they can efficiently detect different sizes of molecules near the inner surface. A theoretical analysis of the sensing capability of microbottle and microbubble WGMRs under different conditions (wall thickness, poloidal curvature, nanoparticle size, *etc.*) is presented in [149]; it has been estimated that about a 10-fold sensitivity enhancement over a microsphere biosensor could be achieved and that nanoparticles less than 20 nm in radius could be detected. Another analysis of the optical properties of microbubble WGMRs, using numerical simulation results based on FEM, has evidenced that, when the wall thickness diminishes to a certain scale (typically around or below 1 μm), the WGMs are dominated by the presence of the liquid core, and the microbubble modes operate in the so-called quasi-droplet regime; this provides an ultra-sensitive way to detect liquid optical properties [150].

**Figure 13:**
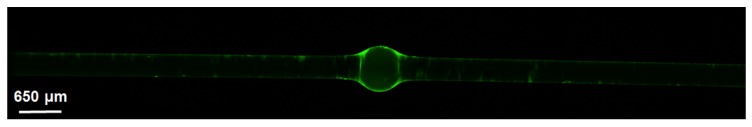
Mosaic image taken by the Zeiss Axio Observer microscope following the sample scan along the microcapillary Z axis. The analysis reveals that the bubble fluorescence intensity is around three-fold higher than that of the microcapillary.

An issue to be carefully considered when using a microbubble resonator for biosensing has to do with the fact that the bubble (or, more generally, the hollow core microcavity) is preceded and followed by the two cylindrical sections of the capillary, which generally have an inner surface well broader than that of the cavity alone. Therefore, if the inner surface of the overall system (microcapillary + microbubble ) is functionalized everywhere and the bioreceptors are immobilized all along the capillary, possible analyte loss may occur during the flow of the solution inside the system, due to undesired bioreceptor-analyte bonds all along the flow path. In order to minimize such undesired bindings, while keeping high the *Q* factor of the microbubble, a spatially-selective photo-chemical procedure has been developed, able to bind fluorescent biomolecules only in correspondence of the WGMR inner surface [117]. The effectiveness of the procedure, which maintains a high *Q* factor (>10^5^ in buffer solution), was proven by fluorescence microscopy and real-time measurement of the resonance broadening. The photo in Figure 13 shows the green fluorescence emission, which is much brighter in the microbubble area than in the capillary sections; here, the bioreceptor is a goat anti-mouse IgG (10 mg/L) labeled with the Alexa Fluor 488 fluorophore, excited at 488 nm. The WGMR brighter zone corresponds to the region where the fluorescent anti-mouse IgG has been covalently immobilized onto the inner surface thanks to the spatially-selective photo-activation of the sulfo-NHS-LC-diazirine cross-linker [117].

## 6. Conclusions and Outlook

Several laboratory experiments have demonstrated the large potential of the WGMR-based sensors in the biomedical and physical fields. WGMR biosensors are at the forefront of today’s sensing technology; a wider exploitation of the unique properties of WGMRs in this area would however require overcoming the still existing difficulties in the integration of the WGMR-coupling system with microfluidics. This is particularly true for microspherical WGMRs, while other WGM resonators, such as microrings and microtoroids, are more easily integrable. In order to exit the laboratories and go to the market, first of all, some packaging problems of the light coupling system have to be solved, as well; robust devices for routine diagnostics with very small sample volumes are necessary. Another needed improvement concerns the complete integration of the sensor and detection in a single chip. This goal would be very important for many other sensing devices, since still, there are few real optical lab-on-chip devices, namely mechanically-stable, reproducible and easy to use devices; rather, we often still have a chip on a lab.

In the last decade, microspherical WGMRs have fully demonstrated their capability of detecting even single molecules, virions, DNA, antibodies, enzymes and aptamers. WGMRs can provide high sensitivity detection of disease markers in point of care testing at the earliest possible stage and high-throughput drug screening. WGMRs are also able to detect larger biological objects, such as bacteria [151,152], thus opening the path to their application in food safety and environment monitoring. Finally, there is a large potential for more fundamental studies in the life sciences, e.g., using WGM resonators for studies of protein folding and membrane biophysics.

## Abbreviations

The following abbreviations are used in this manuscript:

APD	Avalanche photo diode
ARC	Asymmetric resonant cavity
GFP	Green fluorescent protein
LCORR	Liquid core optical ring resonator
MBR	Micro-bubble resonator
MDPI	Multidisciplinary Digital Publishing Institute
OMR	Optical microring resonator
PBS	Phosphate-buffered saline
PDMS	Polydimethylsiloxane
PMMA	Poly(methyl methacrylate)
RIU	Refractive index unit
r.m.s.	Root mean square
TEM	Transmission electronic microscope
WGM	Whispering gallery mode
WGMR	Whispering gallery mode resonator
YIG	Yttrium iron garnet
